# Abnormal 18F-FDG uptakes in the prostate due to two different conditions of urine reflux: a mimicker of prostate cancer

**DOI:** 10.1186/s40064-016-1696-5

**Published:** 2016-01-20

**Authors:** Kensuke Inamura, Yasushi Kaji, Setsu Sakamoto, Akinori Masuda, Takao Kamai

**Affiliations:** Department of Radiology, Dokkyo Medical University, 880 Kita-Kobayashi, Mibu-machi, Shimotsuga-gun, Tochigi 321-0293 Japan; PET Center, Dokkyo Medical University Hospital, 880 Kita-Kobayashi, Mibu-machi, Shimotsuga-gun, Tochigi 321-0293 Japan; Department of Urology, Dokkyo Medical University, 880 Kita-Kobayashi, Mibu-machi, Shimotsuga-gun, Tochigi 321-0293 Japan

**Keywords:** FDG-PET/CT, Prostate cancer, Urine reflux into the prostate, Prostatic utricle cyst

## Abstract

A 69-year-old man with lung cancer underwent 18F-fluorodeoxyglucose (FDG) positron emission tomography (PET)/CT for staging. FDG PET/CT showed high uptakes in the prostate gland with calcification, and magnetic resonance imaging was recommended to check the prostatic malignancy. T2-weighted images revealed midline cystic lesion at the base to midgland level and cystic lesion in right apical peripheral zone. We suspected urine reflux conditions. Voiding cystourethrography demonstrated those cystic lesions were communicating with the urethra. Therefore these lesions were diagnosed as the prostatic utricle cyst and the dilated prostatic duct in peripheral zone. We conclude that the urine reflux condition should be recognized as a prostate benign lesion with FDG accumulation.

## Background

The 18F-fluorodeoxyglucose (FDG) positron emission tomography (PET)/CT scanning has been widely used for cancer staging and assessment of treatment. In prostate cancer, glucose metabolism tends to be higher with increasing Gleason grade, clinical stage and serum prostate specific antigen (PSA) level (Oyama et al. 1999).

However, some benign conditions, show high uptake, because FDG PET/CT findings base on the glucose metabolism, not on cancer-specific features. Representative false positives are inflammatory condition. Adding to this, urine containing FDG may cause false positive results.

In this paper, we present the case with prostatic utricle cyst and dilated prostatic duct, showing FDG uptakes due to urine refluxes to the prostate.

## Case report

**Fig. 1:**
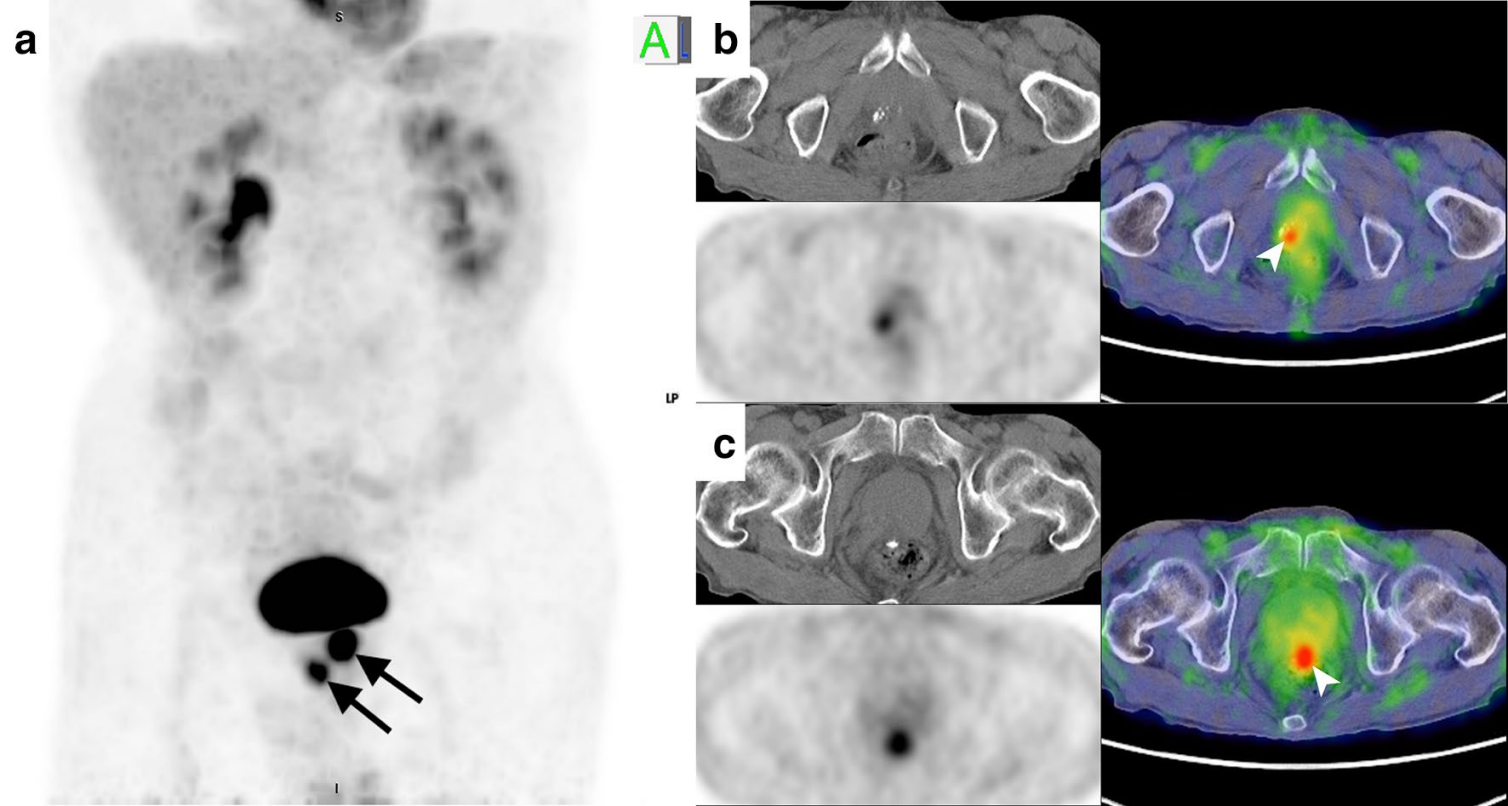
FDG-PET/CT. FDG-PET MIP image (**a**) showed two incidental foci of FDG uptake in the prostate gland (→). On PET/CT, the both lesions at the apex (**b**) and the midgland (**c**) included calcification ()

A 69-year-old man underwent FDG PET/CT for the staging of lung cancer before treatment. His clinical stage was cT2aN1M0, and histologic type was squamous cell carcinoma. FDG PET/CT showed two incidental foci of FDG uptake in the prostate gland. SUV max was 5.1 in the right apex lesion and 8.3 in the midgland midline lesion (Fig. 1). Small calcification was shown in both lesions on CT. He was recommended to investigate the prostatic disorders including prostate cancer.

He was referred to the urology department. At the medical history talking, he complained of difficulty urinating in the morning. Digital rectal examination found no prostate induration and no tenderness. Serum PSA level was 3.91 ng/mL.

**Fig. 2:**
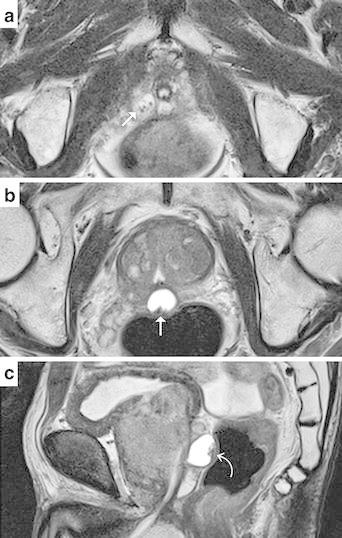
Prostate MR images. Axial T2-weighted images (**a**, **b**) showed two cystic lesions with punctate low signal intensity spot inside (→). Saggital T2-weighted images (**c**) demonstrated the cystic lesion at the midgland level was protruding from the prostate ()

Magnetic resonance (MR) imaging of the prostate was performed for further evaluation. One was located in the right apical peripheral zone and the other was in the medial posterior of the prostate at the base to midgland level (Fig. 2a, b). On axial T2-weighted images, two lesions demonstrated high signal intensity with punctate low signal intensity spot inside. These lesions showed relatively high signal intensity on T1-weighted images. Sagittal T2-weighted images revealed the lesion at the midgland level was protruding from the posterior edge of the prostate (Fig. 2c). Diffusion-weighted images with b value of 1000 s/mm^2^ showed no abnormal high signal in both lesions, suggesting no abscess. After administration of Gd-DTPA (0.1 mmol/kg bodyweight; Magnevist, Bayer Yakuhin, Osaka, Japan), T1-weighted images were obtained and showed no enhancement in both lesions.

We assumed that the cyst in the right peripheral zone at the apex level was the dilated prostatic duct with urinary calculi due to the reflux of the urine, but there was no evidence of the urine refluxing into the prostate in our case.

With regard to the cyst in the medial posterior of the prostate at the midland level, the differential diagnosis of the intraprostatic median cysts are Müllerian duct cyst and prostatic utricle cyst (Shebel et al. 2013). The Müllerian duct cyst is more common in adults while the prostatic utricle cyst often detected in the 1st and 2nd decades of life.

**Fig. 3:**
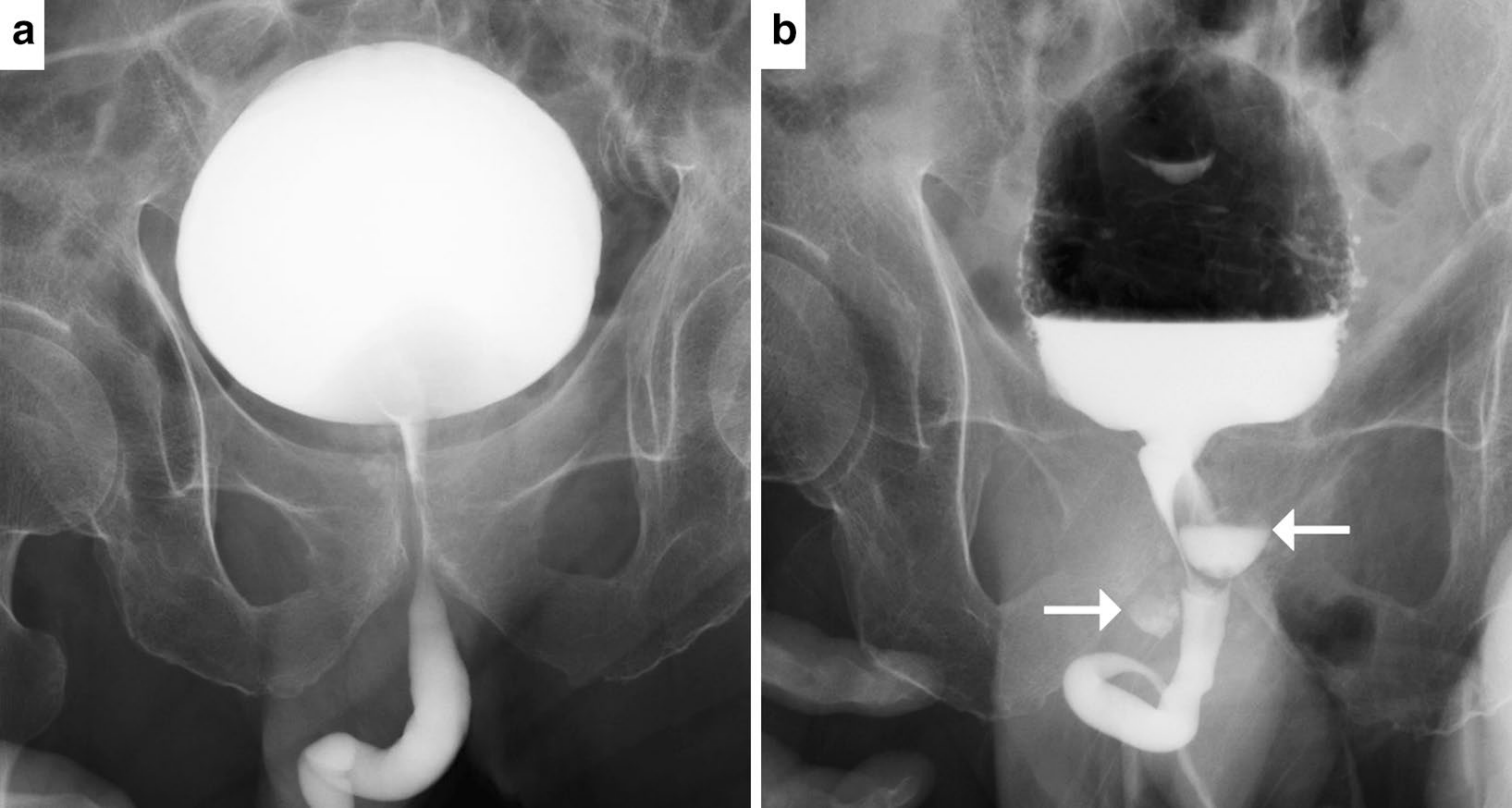
Voiding cystourethrogram. The early voiding phase (**a**) showed no significant abnormal findings. The late voiding phase (**b**) demonstrated contrast media in urethra was filled into those prostatic cystic lesions (→)

To explain those calcification depositions and FDG uptake, the existence of the prostatic utricle cyst or communication with prostatic urethra was reasonable. Voiding cystourethrography was performed additionally and it demonstrated that the contrast media in urethra was filled into those prostatic cystic lesions (Fig. 3a, b).

With these findings, the urine refluxing to the dilated prostatic duct and the prostatic utricle cyst was proved. FDG uptakes in the prostate were not owing to prostate cancer but to the urine reflux to the prostate.

## Discussion

It is known that the incidental focal FDG uptake lesions in the prostate are not always indicated cancer. Although it is controversial, some researchers suggest FDG uptake to the prostate cancer depends on its size and pathological features, such as Gleason score and degree of differentiation. On the other hand, it have also been reported that benign lesions, such as prostate hyperplasia (Hoh et al. 1998; Lawrentschuk et al. 2006) or prostatitis (Kao et al. 2008) can show FDG uptake.

The FDG uptakes in our case could not be explained fully by these above reasons. And the strong accumulation of FDG to cystic structure seemed strange. Then we considered the existence of the prostatic utricle cyst or communication with prostatic urethra, which brought the urine refluxing into the prostate.

Intra-prostatic urine reflux is considered one of the etiological factors in bacterial prostatitis. Kirby et al. (1982) demonstrated instilling carbon partible suspension into the bladder, and revealed the urine reflux with macroscopic and microscopic evidence of carbon in the prostatic duct. In our case, the cystic structure in the right peripheral zone at the apex level could be considered the dilatation of prostatic duct due to recurrent inflammation.

The prostatic utricle cyst is an embryologic remnant of the Müllerian duct system, resulting in incomplete regression of this structure during embryologic development (Shebel et al. 2013). Radiologically, it does not extend above the base of the prostate, which communicates freely with the prostatic urethra. These findings are important to distinguish from the Müllerian duct cyst. The cyst in the medial posterior of the prostate at the midland level has these findings and is compatible with prostatic utricle cyst in this case (Shebel et al. 2013).

Prostate calculi are predominantly found in the larger prostatic ducts and acini of the posterior to lateral lobes of the prostate, especially in the transition zone rather than other zones (Suh et al. 2008). Those calculi within the ducts are relatively larger and visible grossly than those within the acini. Some researchers assumed that prostate calculi are formed by the deposition of calcareous material in corpora amylacea due to the intra-prostatic reflux of urine (Kirby et al. 1982; Nickel 1999). The reason why calculi in transition zone are more common is probably because corpora amylacea are predominant in the transition zone of the prostate.

With the growing calculi itself, or together with the inflammation or benign prostate hyperplasia, occlusion of the other acini occurs and results in further deposition of calcareous material, leading to concentric calcification.

Han et al. (2010) and Seino et al. (2014) mentioned calcification in the presence of focal FDG uptake in the peripheral zone of the prostate was associated with benign lesions, while peripheral zone subcapsular foci with no calcification were more likely to be malignant. Our case can explain the relationship between benign focal FDG uptake and calcification, under the situation of the urine refluxing into the prostate.

The urine refluxing into the prostate can be the reason of the FDG-avid lesion besides the hyperplasia or inflammation, and the calcification in the prostate would be seen in this situation.

In our case, MR imaging showed the FDG-avid lesions were cystic lesions, and clearly depicted those morphological features or positional relationship between the cystic lesions and surrounding structures.

## Conclusions

We experienced FDG-avid lesions in the prostate caused by reflux of urine into the prostate. We should recognize the urine reflux condition as a prostate benign lesion with FDG accumulation.

In such situations, we suggest to review the CT images of PET/CT. If CT cannot depict a cystic part with calcification in corresponding prostate area, MR imaging is also helpful to evaluate these lesions besides serum PSA level and ultrasonography.
